# Extremist identity creation through performative infighting on Steam

**DOI:** 10.3389/fpsyg.2025.1586566

**Published:** 2025-08-12

**Authors:** Alex Bradley Newhouse, Rachel Kowert

**Affiliations:** ^1^Department of Political Science, University of Colorado Boulder, Boulder, CO, United States; ^2^Department of Psychiatry, University of Cambridge, Cambridge, United Kingdom

**Keywords:** video games (psychology), extremism, social network analysis, far-right, social media

## Abstract

The video game marketplace Steam has long hosted a lively social network for the purpose of connecting game players and game developers. Over the past 5 years, however, neo-fascist and neo-Nazi communities have begun using Steam's community features to build large-scale socialization and identity creation networks. These networks, while insular, involve large numbers of Steam groups and users, who share hateful and violent content with one another. In addition, these same users frequently spread extreme messages on more public-facing content, including in game reviews and game forums. Using open-source data and scaled social network analysis, we show that the far-right ecosystem on Steam possesses characteristics of collective radicalization and mobilization. This poses both an immediate danger to gamers and game developers who rely on Steam and also a longer-term risk to social safety.

## 1 Introduction

Since its launch in 2003, Steam has become the dominant marketplace for purchasing video games on PCs. Taking advantage of the decrease in physical game sales (e.g., on CDs, DVDs, or Blu-Rays), Steam's developer, Valve, has built the platform into a behemoth and has established it as the most popular place to buy and launch PC games. While accurate and comprehensive metrics are scarce, most measures suggest that Steam has tens of millions more monthly active users and earns much more money than its nearest competitors (Strickland, 2022).

While Steam's storefront is its core feature, the platform also provides a number of different social features and and functions for both game players and game developers. Developers are able to run dedicated forums for their games and communicate directly with their community of players; users, meanwhile, can leave user reviews, write in a game's forums, and participate in the broader Steam Community ecosystem. Steam's popularity and these features have allowed Steam to transform into a large, popular social media platform in its own right. The complex dynamics of Steam's social features have led developers to dedicate community management resources to maintaining and improving their presence and relationships with players on Steam.

However, Steam's forums, user reviews, and community features have experienced large volumes of adversarial conduct from users. This conduct includes toxicity, hate speech, harassment, coordinated brigading and “review-bombing,” and other anti-social behaviors. Valve rarely comments on its content moderation practices and, when it has, it has historically defended a light approach to content moderation (Valve Corporation, 2018). This has led to significant backlash and criticism from developers and targeted users, who have argued that Steam is becoming a sanctuary for bad actors.

In this paper, we explore one set of dangerous users particularly active on Steam: far-right extremists, specifically neo-fascists and white supremacists. Using scaled tools to gather social network data of neo-fascist activity on Steam and employing both quantitative and qualitative analysis to understand their dynamics, we show that Steam is currently playing host to widespread identity creation and performative infighting, behaviors important in the development of far-right extremist networks. We demonstrate that users in this neo-fascist ecosystem transform their ideologies via propaganda rapidly and continually, and that they hide “in plain sight” by masking virulent antisemitism and hate speech with in-group language.

Steam has received increasing attention from researchers and analysts in recent years, but it has largely been passed over by social media researchers in favor of a focus on bigger or more obviously antagonistic platforms. In this paper, we argue that researchers should devote more time and effort to understanding and studying the Steam's particular dynamics.

## 2 Background

### 2.1 Steam as a social network

The importance of Steam in the games industry has inspired a thriving body of sociological, ethnographic, and information science literature investigating community dynamics and player behavior. Steam's primary purpose is to provide a marketplace and library where people can purchase and play video games on PCs. When an individual creates an account, they are directed to Steam's storefront. Social features are backgrounded, either embedded in game pages (user reviews, specific game discussion boards) or hidden in Steam's community tab (user groups and general forums). As a result of this, Steam's function as a social network is somewhat unusual compared to other purpose-built social platforms, with games providing a unique lens through and around which relationships are built.

Steam's various social functions are, for the most part, siloed from each other, with only a few inter-function connections (game pages to reviews, and game pages to forums). Steam is primarily unified by connecting all functions to a user's profile page. Steam users can promote any Steam function they use directly from their profile, including any games they play and own, groups of which they are members, their friends lists, their own artwork and any artwork they like, and their collection of in-game badges and achievements. Steam users can also enable a feature that lets other people comment on their profile.

The social graph outside of the games marketplace, consequently, is dependent on user profiles for connectivity, discoverability, and functionality. Users drive the creation of user groups, which function similarly to Facebook groups. While there is no native functionality on Steam for groups to interact directly with one another, informal group-level collaborations, conflicts, and connections often arise. Additionally, because there is no cap on the number of groups of which a user can be a member, groups can be seen as “clustering” with one another based on large overlaps in membership.

In one of the initial large-scale attempts to characterize the Steam community, Becker et al. (2012) scraped information for every public account, group, and game (at the time of writing in 2011, this totaled 9 million accounts). The authors find that Steam's network became increasingly clustered between 2008–2011, indicating users are increasingly attracted to similar users and insulated from the broader network. O'Neill et al. (2016) also characterizes the platform's entire community; writing 5 years later than Becker et al. (2012), the authors collect data on over 108 million accounts and 384 million owned games. This study finds that Steam's community dynamics have particularly long tails across a variety of metrics, including games owned, playtime, and friendslist size. The authors find additional evidence that users tend to cluster together based, in part, on these metrics.

Loria et al. (2021), meanwhile, finds that Steam friendships are generated in the context of specific games, but that these relationships can be characterized better by collective decisions than by the games alone. User-generated tags are more descriptive of friend clusters than game titles, leading the authors to conclude that user perceptions of games are linked to and reciprocal with their perceptions of their communities. Li and Zhang (2020), too, find that Steam tags are important for collective dynamics, showing that group identities can be characterized by the networks organized around certain tags.

Despite Steam's centrality in organizing communities of video game players, anti-social, toxic, and extremist behaviors have only rarely been explored in the scholarly literature. Most of the existing research focuses on collective toxicity, especially the act of “review bombing,” where a network of individuals express grievances with a product through the coordinated and short-term publishing of a high volume of negative user reviews. Designed to artificially deflate review scores, review bombing has been observed across numerous marketplaces (Tomaselli et al., 2021).

The first systematic review of extremist behavior on Steam was presented in Anti-Defamation League (2024). Researchers at the ADL's Center on Extremism conducted a near-comprehensive analysis of Steam user profiles, groups, and interactions between users, finding a high volume of explicit antisemitism, neo-Nazism, and other forms of hateful content. This study highlights the frequent use of copycat content as well as the prevalence of extremist-aligned visual imagery in profile pictures and user-generated content. Its findings imply that Steam is commonly used for the establishment of networks of hardcore extremists, as the expanse of highly virulent content that Anti-Defamation League (Anti-Defamation League (Anti-Defamation League (Anti-Defamation League (Anti-Defamation League (Anti-Defamation League (2024) discovered is most likely not aimed toward radicalizing newcomers. The publication of this report appears to have induced Valve to more widely ban explicit neo-Nazi and white supremacist behavior, but extremist cliques and groups are still easily discoverable across Steam's social features.

### 2.2 Collective identity creation in extremist networks

This paper aims to investigate extremist behavior on Steam within the framework of collective identity formation. Tracing back to the social identity theory tradition pioneered in Tajfel and Turner (1978) and Tajfel (1979), studies of collective identity creation suggest that interactions between people can produce a sense of identity influenced by, but distinct from, individuals' sense of self. Collective identity has long been identified as an important factor in the development of social movements and political behavior; it has been a useful concept in bridging gaps between social movement theories, such as group psychology and resource mobilization (Fominaya, 2010; Polletta and Jasper, 2001).

As a result, collective identity has been increasingly used as a method of understanding extremist movements. Extremism, which we take to mean a belief that one or many out-groups pose an existential threat to an in-group requiring hostile action in response (Berger, 2018), is generally a collective activity, developed via relationships to others in a close network (Berger, 2017). As part of the process of developing a collective extremist identity, individual actions are often geared toward signaling commitment, camaraderie, and shared goals to other members of the in-group (Smaldino, 2022; Rasoulikolamaki et al., 2023; Glaeser and Sunstein, 2009). In this paper, we focus on engagement with *far-right extremism* specifically, defined as a broad milieu of antiegalitarian, antidemocratic, and white supremacist ideologies. Because of the particular sample of users and groups that appear to be particularly active on Steam, we explore behavioral patterns among users espousing fringe elements of the far right, such as neo-fascism (Cento Bull, 2010; Upchurch, 2021), apocalyptic millenarianism (Wilson, 2012; Newhouse, 2021), and eliminationist white supremacy (Belew, 2018).

As Internet spaces become more central to extremist movements throughout the world, radicalization and mobilization dynamics have subsequently been transformed. Geographic and organizational decentralization have replaced conventional hierarchies and command and control structures, resulting in the development of bottom-up and reciprocal trends in radicalization. These trends generally depend on the creation of increasingly intense collective identities. For instance, the so-called “Saints culture,” wherein large-scale online communities have developed a process of canonizing and deifying mass shooters, has been described as possessing a “cumulative momentum” driving participants toward extremism, despite no clear membership, leadership, or strategic goals (Macklin, 2022). Antinori (2017), meanwhile, identifies a similar pattern among digitally active jihadist communities, describing the “Swarm Wolf” as a seemingly lone-actor attacker who is actually the product of a global ecosystem of collectively radicalizing extremists. Finally, Fürstenberg (2022) and Lee and Knott (2022) argue that online communities of neo-fascists develop “collective learning” dynamics that train participants in the identity, history, and goals of the neo-fascist milieu.

Video games and game-adjacent spaces (Schlegel and Kowert, 2024) may be particularly adept at catalyzing these collective identity and cumulative radicalization processes, even compared to other social media platforms. Kowert et al. (2022) extends the concept of *identity fusion* from social psychology to games studies, showing that multiplayer video games generate spaces that facilitate rapid, and potentially dangerous, fusion of an individual's personal identity with that of their (friend, teammate, etc.) group. Other studies have revealed activity in video games like Minecraft and Roblox indicative of collective radicalization, mobilization, and exploitation by extremists (Newhouse and Kowert, 2024; Koehler et al., 2023).

Game-adjacent spaces that provide services to run alongside games provide additional opportunities for social processes pushing toward identity fusion and radicalization. For instance, case studies have investigated the messaging app Discord (Davey, 2024; Schlegel, 2021) and the live-stream platform Twitch (Schlegel, 2021; Lankford et al., 2024), among others. These studies have demonstrated that extremist activity crosses over between games and these complementary services, suggesting that collective processes in relation to games occur in multi-platform contexts.

In this paper, we focus on the case of Steam to illustrate how collective identity creation dynamics in extremist movements occur on a game-adjacent platform. We introduce the concept of “performative infighting,” defined as the symbolic performance of hostility within an in-group. In particular, we are interested in the strategic adoption of theatrics that appear on the surface to indicate sectarianism, ideological conflict, or intragroup friction, but in reality are merely superficial.

This concept builds on contentious politics literature, which has studied the role of infighting in social movements. Historically, infighting has been viewed as an intrinsically destructive phenomenon within movement contexts, complicating the ability of movements to mobilize resources and direct membership toward goals (McAdam, 1999; Gamson, 1995; Zald and McCarthy, 1987). This is particularly true of studies of extreme right-wing movements, and activists hoping to disrupt these movements have attempted to spark infighting through infiltration and other means (Ince, 2011; Ramalingam, 2015). However, a growing body of literature has complicated this characterization, suggesting that competition and conflict within movements can help them construct collective identities and formalize strategic goals (Ghaziani and Kretschmer, 2018). This complements studies of online trolling, which suggest that the process of trolling provides a constitutive effect for online communities transitioning to political activism (Phillips, 2015).

Here, we contribute to the study of movement infighting by relaxing the assumption that infighting is always “organic,” or engaged in by people with “real” disagreements. We suggest here that some types of infighting are done performatively, whereby participants adopt the aesthetic trappings of intra-group conflict, with little true hostility meant between “belligerents.” We show how this works to bind a cluster of Steam activity together, facilitating the development of an extreme-right collective unique to the Steam platform. Moreover, we show how this collective is constituted around and in relation to video games, contributing to the understanding of the phenomenon of “extremism gamification” (Schlegel and Kowert, 2024).

## 3 Methods

Due to the highly decentralized, fast-transforming nature of online ecosystems, analysis of behavioral patterns on a network level can provide a more nuanced understanding than other methods (Newhouse, 2021). In this paper, we focus on the informal dynamics of user-group and inter-group relationships on Steam. At the lowest level, our social network analysis is based on the functional ties formed by a user's membership status in a set of Steam groups. Further, we generate an inter-group network by assigning weighted ties between two groups depending on the number of members they share.

Steam's social functionality is focused on the user as the core actor. Users are represented by a username attached to a profile, a dynamic webpage that provides an overview of their Steam activity. Users can customize their profile page to showcase different elements; common characteristics include most-played games, trophy cases, user-generated content like art and in-game images, friends lists, group lists, items, and a “wall” on which other users can leave messages.

Users can interact with each other through several different social functions. Most commonly, users will become friends with one another, direct message each other, and invite each other to play games. Users can also interact in community forums, which include both game-specific boards and general boards organized around topics. Finally, users can join groups, similar to Facebook groups, that have their own pages and provide some tools for group events and organization.

In contrast with other studies of extremist activity on Steam, we focus on collecting relational data in addition to user- and group-level information. We collect this data by building a tool for crawling Steam's XML trees, which surface information for both group membership (who is in a certain group) and user participation in groups (in which groups is a particular user).

We build a bipartite (two-mode) network consisting of user-group relationships via snowball sampling, a popular method for iteratively gathering relational data when initial access to samples from the population of interest is limited (von der Fehr et al., 2018; Leighton et al., 2021). We begin our snowball sample with a single Steam group that the authors identified during manual investigation. Its name, profile picture, and group page include symbols and language strongly linked to eco-fascism (Hughes et al., 2022).

We expand our social network by first gathering all members of this seed group, creating an initial sample of 173 users. We then gather the names of all other groups of which these users are members, generating the one-step ego-network of our seed group. Next, we collect all additional users that have membership in this new set of groups. This results in a network composed of 11,591 groups and 3,379,330 users.

While the relational data alone can reveal important information about collective behavior and identity formation, the characteristics of each node in the network—each distinct Steam group—is vital for characterizing the roles of different structural elements. As such, we also gather metadata for each group, including name, summary, and member count, and each user, including username and profile description.

## 4 Ego network exploration

Our first Steam group, which promotes eco-fascism and antisemitism in its group homepage, has 173 members. The metadata of these users allows us to illustrate some shared dynamics of Steam's far-right extremist audience. One method of understanding this network is by investigating Steam's “Most Played Games” data field, which captures a user's highly played games that they choose to highlight on their profile. Games appear in rank order by the number of hours spent in them; its prominent place on profile pages provides an opportunity for signaling preferences to other users. We show the most common games that appear in the Most Played Games rankings in Table 1.

**Table 1:** Common games in most played rankings on Steam profiles of 173 members of eco-fascist group.

**Game title**	**Number of occurrences in network**
Counter-strike 2	71
Hearts of iron IV	59
Garry's mod	42
Dota 2	18
Team fortress 2	18
War thunder	18
Wallpaper engine	15
Grand theft auto V	13
Arma 3	12
Helldivers 2	12
Half sword playtest	12
People playground	12

While most of these games are commonly played among all types of users, Hearts of Iron IV (HOI4) is an important exception. HOI4 is a strategy game developed by Paradox Interactive in 2016, and it was originally played primarily by hardcore strategy gamers. However, since its launch, neo-Nazi and neo-fascist communities have increasingly adopted it as a symbol. Far-right communities have embraced the ability to play as Nazis and fascists in HOI4, and they have particularly celebrated the ability to create speculative fiction via the built-in story and user-created mods (White and Lamphere-Englund, 2024; Newhouse and Kowert, 2024).

Users in this network are also active in using other elements of their profiles to signal to one another. Steam usernames, for instance, often contain references to fascist terminology, text versions of fascist symbols, or explicit antisemitism and hate. Among this set of users, we identify multiple instances of swastikas, Nordic runes affiliated with white supremacist movements, and common Nazi terms like “88” and “GTK” (see Table 2).

**Table 2:** Common symbols in Steam usernames in eco-fascist network.

**Term, symbol, or slogan**	**Frequency**
Wolfsangel	7
Algiz rune	7
Iron cross	7
Odal rune	5
88	2

These 173 users are themselves members of over 11,000 Steam groups beyond our initial starting point, although this distribution is highly skewed. The top four users each are members of over 2,000 groups, but the median user is only a member of 52. In addition, this particular segment of the Steam userbase is highly densely connected, with close neighborhoods of users and groups numbering in the thousands.

**Figure 1:**
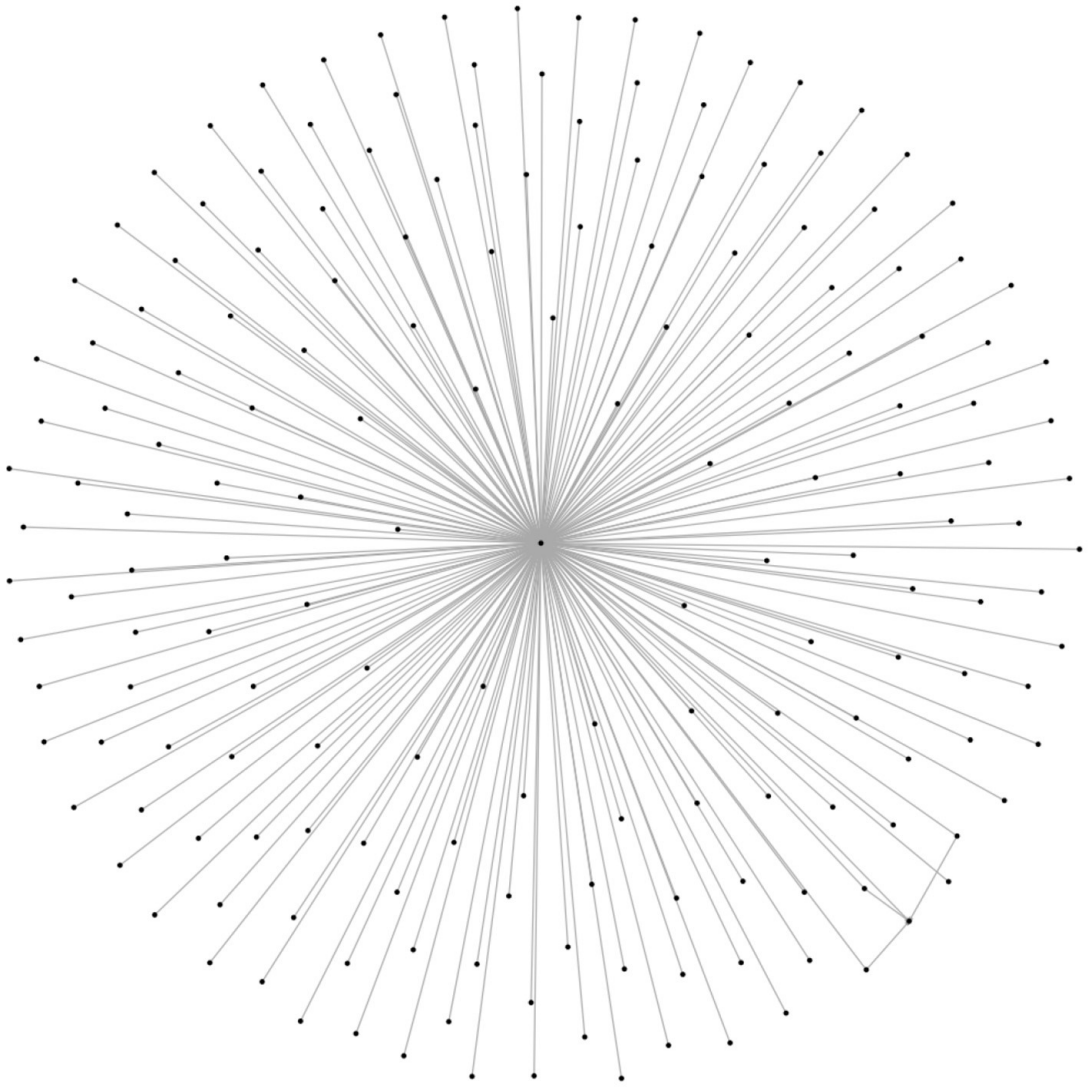
One-step ego network around our seed group, which espouses eco-fascism and other forms of far-right extremism. As we are studying a bimodal network, this depicts the seed group connected to each of its members.

**Figure 2:**
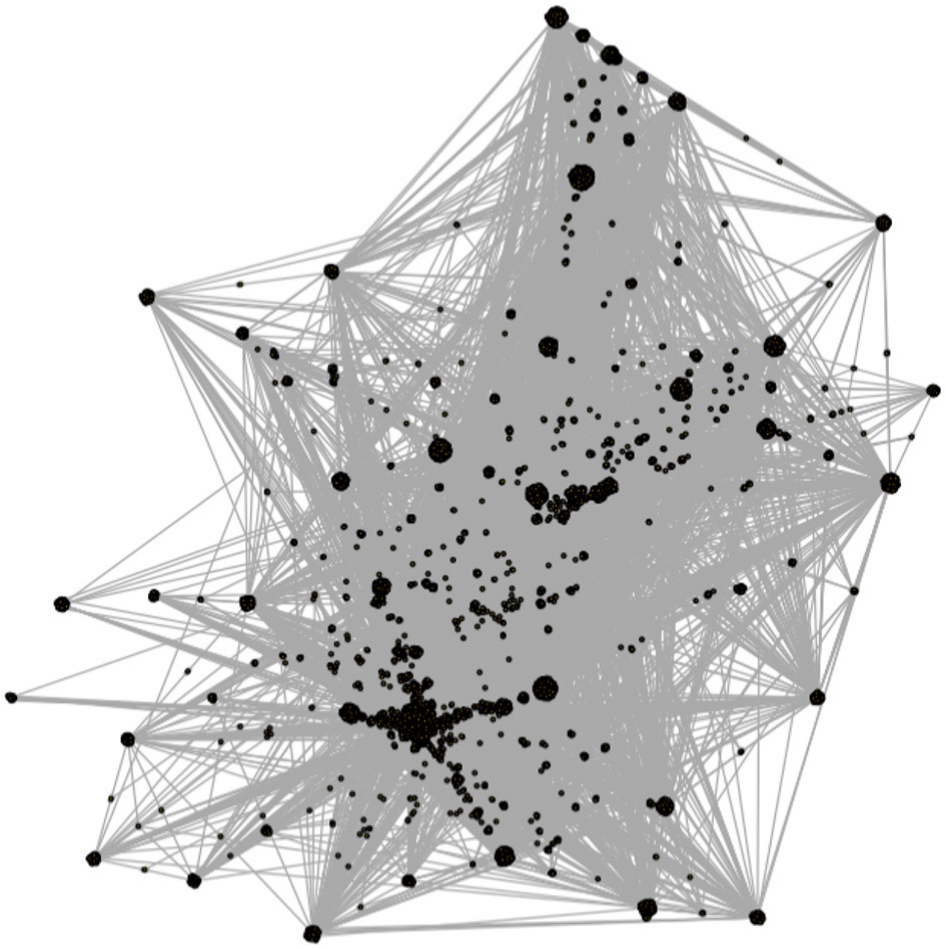
Two-step ego network around our seed group. This visualization includes our seed group, each of its members, and each additional group those users are members of. This visualization depicts how densely connected the far-right community is on Steam. Groups that share large numbers of members are commonplace, which results in the users and groups (black dots) being pulled close together in the visualization.

To illustrate these relational dynamics, we visualize parts of the overall network by showing the progression of snowball sampling from the seed group. Figure 1 shows the “one-step” ego network, in other words the network composed of our seed group (the middle node) and its members (every node on the periphery). Figure 2 expands this view to the seed group, its members, and all other groups those users are members of. Despite only going one step further out from the seed group, this network adds a huge amount of complexity and scope; most notably for our purposes, it also shows a high level of overlap between Steam groups, resulting in numerous and often quite short paths between different parts of the overall community.

Of course, because our sample exponentially grew in scale after only a couple of sampling steps, many of these groups in the periphery may not be extremist-aligned and are likely normal, mundane groups of game players. We can identify potential extremist-aligned groups via member overlap analysis. We select the ten groups containing the highest overlap with the original seed group, with common members ranging from 28 to 60. All ten of these groups contain immediate and explicit white supremacist, neo-Nazi, or other far-right extremist symbols and terminology. These signals are variously contained within group names, headlines, and descriptions. For instance, one Steam group surfaced through this method has a name referencing Varg Vikernes, a notorious neo-Nazi musician. Another openly promotes “techno-fascism,” while a different group declares 9/11 conspiracy theories in its headline.

**Figure 3:**
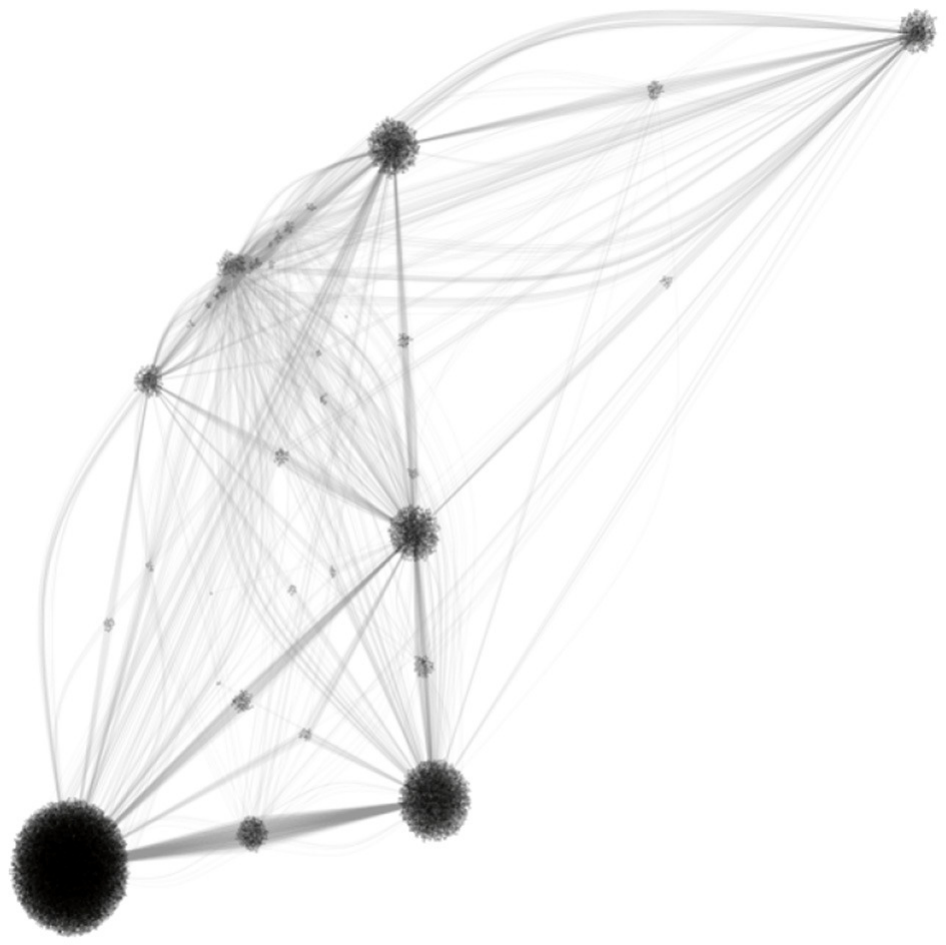
High-level visualization of user-group network for seed group and its 10 most similar neighbor groups. This is a bimodal visualization, showing tightly connected cliques bridged by shared membership, depicting how even clusters of similar groups can act as constituent parts of a larger ecosystem.

Figure 3 shows a high-level view of how our seed group and its 10 most similar groups (by number of shared members) are connected to one another. Groups are identifiable by their tight clusters of members (making them look like pin cushions), while bridges between these “pin cushions” are created by overlapping members. These bridges drive the creation of the overall community, providing potential avenues for information dissemination, cross-collaboration, and other relational dynamics.

## 5 Performative infighting sub-network

During manual review of some of the highly overlapping Steam groups in this network, we identified a sub-network of users and groups that engage in an activity we describe as *performative infighting*. In particular, a cluster referring to itself by a common name^1^ claims to be experiencing one or multiple “wars,” fought by Steam groups in the cluster. These Steam groups have arrayed themselves into digital battle lines, whereby members and groups align themselves into “ally” and “enemy” relationships. Ally lists almost entirely include other Steam groups in the cluster; entities listed as “enemies” include both other Steam groups and common villains in far-right extremism, such as the Anti-Defamation League and populations of individuals in rival communities.

We identify these characteristics because a subset of the groups involved declare these allegiances and rivalries in their Steam descriptions, creating informal network ties that delineate the axis of contestation. While it is unclear exactly what the nature of the battle is, far-right ideologies and symbols are represented on both sides.

This cluster includes at least 40 distinct Steam groups of varying levels of activity. Some appear to have been dormant for the past several years; others have been updated and posted announcements during the time we have been monitoring them. The groups in this cluster range in size, but most have between 100–200 members and are densely connected. Using the same snowball-sampling method as before, we find that the 215 members in one of the “hub” groups in this cluster tend to share high numbers of groups in common with one another. For example, 12 groups share at least 40 members in common with the hub group.

Group names, group descriptions, and member profiles indicate that this cluster promotes a particularly fringe set of ideologies. Several group names suggest knowledge of and admiration for Order of Nine Angles and Tempel ov Blood, particularly violent networks of neo-fascists that have engaged in crimes such as terrorism, ritual murder, and child exploitation (Upchurch, 2021; Shah et al., 2023). Other group names are indicative of links to the Trollwaffen network, a loosely affiliated band of white supremacists, neo-fascists, and other extremists defined by its commitment to adversarial Internet behavior, coordinated harassment, and frequent evasions of platform bans (Newhouse and Kowert, 2024). Still others, discovered via use of the Internet Archive's Wayback Machine, explicitly endorsed the child exploitation- and terrorism-linked Com network (Argentino, 2025).

### 5.1 Transformations over time

The sub-network's emphasis on waging digital “wars” has been occurring since at least 2022, based on archival review we conducted using the Internet Archive's Wayback Machine. Alignments between the groups have periodically changed, with some groups switching from alliances to enemies (and vise versa), while other groups have been declared “defeated,” seemingly as a result of bans by Steam's content moderation team.^2^ One extant group at the time of this writing, which serves as one of the primary hubs for publicly declaring alliances and rivalries, has gone through at least eight different versions in response to bans. This group previously appears to have declared its ban evasions as “wars won,” including this count as part of its group description.

**Figure 4:**
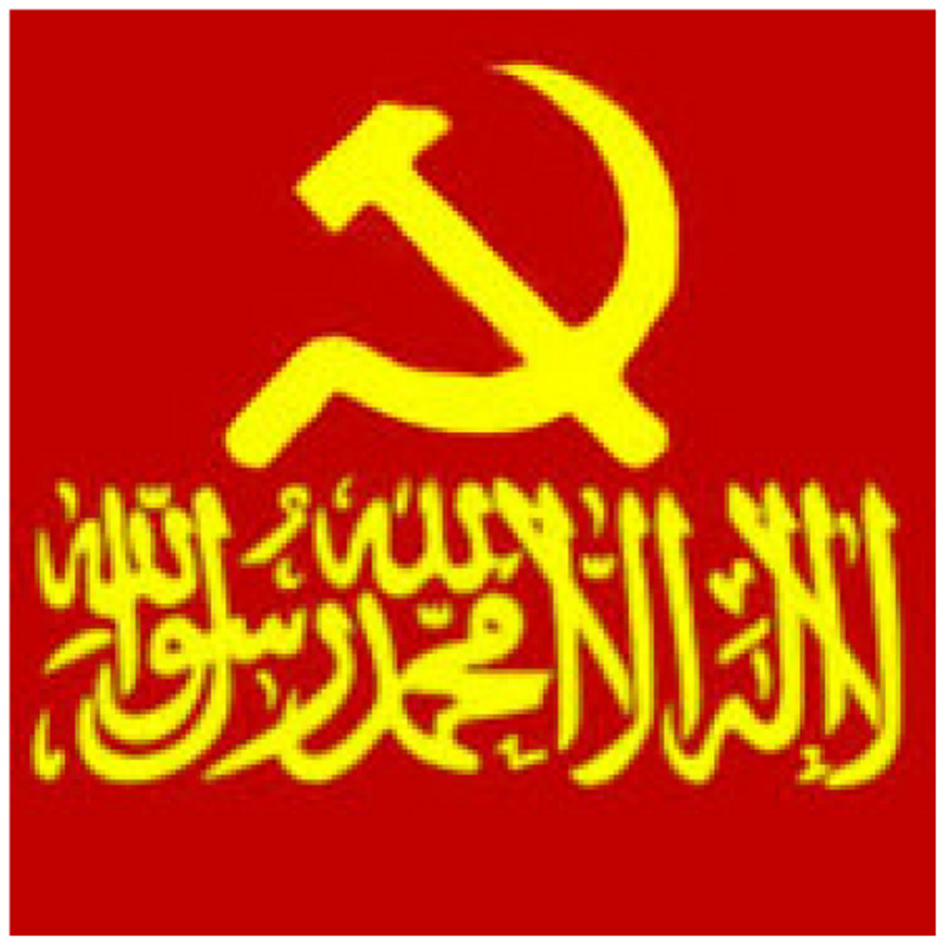
Picture used by now-banned Steam group that was involved in performative infighting. This profile picture represents memeified syncretism: common among extreme-right networks is the semi-ironic mixing of different ideologies; in this case, the Islamic State flag is remixed with the Soviet hammer and sickle.

Group pictures and aesthetics have also changed over time, likely in response to a combination of Steam moderation pressure and developments in the cluster's “wars.” In 2022, for instance, several groups in the network used far-right symbols as their group pictures. Figure 4 shows one particularly notable example from a now-banned group, which combines the aesthetics of the Islamic State with that of communist totalitarian regimes. This blending of symbolism from seemingly contradictory ideologies is a trademark of online neo-fascist communities, which often emphasize ideological syncretism and the remixing of extremism into new aesthetics for neo-fascism (Argentino et al., 2022).

## 6 Discussion

Behavioral indicators suggest that this effort to play-act a sectarian war was likely intended to (1) publicize fringe ideologies and networks to the broader Steam community, (2) build resilience to content moderation action from Steam, and (3) define the boundaries of in-group and out-group.

First, many users participating in this performance of infighting intended to spread awareness of groups in the cluster. In the most active “hub” group which still maintains a list of all allies and neutrals (declaring all enemies vanquished), users frequently share links to other like-minded Steam groups and also call fellow members to invite more people in. “The official [name redacted] steam group has just surpassed this group in members,” a recent announcement declares. “Are we gonna let that [expletive] slide?” In November 2024, an administrator with an antisemitic username wrote, “Whoever invites the most people gets admin and player of the week.” Many other similar posts across the cluster cross-promoted groups, shared links to external networks, and otherwise attempted to market this cluster to other places within Steam.

Second, this infighting and networking effort is likely intended to provide increased resilience to occasional waves of moderation from Steam itself. By building alliances and enemy lists, and by cross-promoting the Steam groups so extensively, this performative infighting produced a network highly capable of reformulating if certain nodes in the network were to be banned. Indeed, as previously mentioned, one of the main hub groups in the network has re-launched at least 8 times with variants on the same original group name. As one member described the dynamic at play in 2023, “You can kill the group, but can't kill the idea... JOIN THE [group name] LIVES ON or was it [variant of group name]? I don't remember BUT ANYWAY LIVES ON.”

Despite bans applied to many of the groups explicitly endorsing the Com cybercrime network, Steam's content moderation appears to have been inconsistent and not comprehensive, resulting in many opportunities for restructuring and reformulation. As part of the performative infighting, several groups have kept a running tally of the allies “lost” and enemies “vanquished” as a result of these ban waves. This provides an easy-to-access digital Rolodex of surviving neo-fascist communities to join.

Finally, the performative infighting serves to help this network of groups and users define the boundaries of in-group and out-group. The most extensive alliance list still active, numbering at around 40 Steam groups, defines the foundational collective of this particular ecosystem of online neo-fascists. Enemy lists, meanwhile, can indicate both long-term villains of the movement (like the Anti-Defamation League) as well as sets of users that are temporarily exiled, disliked, or simply being trolled. For instance, in one enemy list from 2023, the Steam moderation team is included alongside the Bureau of Alcohol, Tobacco, and Firearms as well as several Steam groups from adjacent networks in the extremist ecosystem.

## 7 Conclusion

In this paper, we introduce the concept of *performative infighting* to describe a phenomenon that occurs in online extreme-right milieus. We argue that performative infighting combines the effects of online trolling and “constructive” infighting to produce the conditions for collective identity formation and mobilization.

To illustrate this concept, we collect and analyze open-source social network data from the video game marketplace Steam, a “game-adjacent platform” that is particularly influential in the games industry. Focusing on Steam user-group relationships, we build a two-step ego network around our seed node, a Steam group that publishes explicitly eco-fascist content. Through manual investigation, we identify a sub-network composed of at least 40 Steam groups and at least several hundred Steam users that have been constructing a satirical, mostly fake “war.” This performative infighting includes extensive use of neo-fascist imagery, promotion of antisemitic content, and frequent slurs, insults, harassment, and other toxic behaviors. Through our descriptive analysis of this sub-network, we argue that such performative infighting provides an extremist milieu with an increased marketing reach, resilience to moderation, and the ability to more clearly define in-groups and out-groups. In other words, this cluster of activity has indicators of collective mobilization and radicalization.

This paper provides important insight into the methods by which extremist communities exploit games and game-adjacent platforms. Specifically, in addition to more conventional radicalization and recruitment activities, the Steam network we investigate displays highly flexible, personalized, and decentralized behavior. This emphasizes the need for scholars of games, extremism, and social psychology to expand the scope of studies on games and extremism. Rather than narrowly focusing on specific ideologies or groups and their interactions with video games, platforms should be studied with an eye toward tracking artifacts of extremism wherever they appear. We propose that this bottom-up method of studying games and extremism will lead scholars to more adaptable conclusions and, potentially, policy interventions.

## Data Availability

The raw data supporting the conclusions of this article will be made available by the authors, upon reasonable request.

## Footnotes

^1^To prevent incidentally popularizing this network, which contains extreme and violent users, we have redacted all identifying information.

^2^While the overarching cluster has mostly remained intact, some groups have periodically been banned for violations of community rules.
